# A Review of Molecular-Level Mechanism of Membrane Degradation in the Polymer Electrolyte Fuel Cell

**DOI:** 10.3390/membranes2030395

**Published:** 2012-07-10

**Authors:** Takayoshi Ishimoto, Michihisa Koyama

**Affiliations:** 1 INAMORI Frontier Research Center, Kyushu University, 744 Motooka, Nishi-ku, Fukuoka 819-0395, Japan; Email: ishimoto@ifrc.kyushu-u.ac.jp; 2 International Institute for Carbon-Neutral Energy Research, Kyushu University, 744 Motooka, Nishi-ku, Fukuoka 819-0395, Japan

**Keywords:** polymer electrolyte fuel cell, perfluorosulfonic acid membrane, chemical degradation, molecular level analysis

## Abstract

Chemical degradation of perfluorosulfonic acid (PFSA) membrane is one of the most serious problems for stable and long-term operations of the polymer electrolyte fuel cell (PEFC). The chemical degradation is caused by the chemical reaction between the PFSA membrane and chemical species such as free radicals. Although chemical degradation of the PFSA membrane has been studied by various experimental techniques, the mechanism of chemical degradation relies much on speculations from *ex-situ* observations. Recent activities applying theoretical methods such as density functional theory, *in situ* experimental observation, and mechanistic study by using simplified model compound systems have led to gradual clarification of the atomistic details of the chemical degradation mechanism. In this review paper, we summarize recent reports on the chemical degradation mechanism of the PFSA membrane from an atomistic point of view.

## 1. Introduction

The polymer electrolyte fuel cell (PEFC) has attracted much interest as a promising power source for automobiles and cogeneration systems because of its high energy conversion efficiency with environmental benefits. The PEFC system has already been commercialized for residential use under the common name of “Ene-Farm” in Japan in 2009 [1,2]. For larger penetration of PEFC into society, practical long-term operation with a drastic cost reduction is necessary. One of the problems is the durability of the membrane electrode assemble (MEA) [3,4,5,6,7,8,9]. Degradations of the Pt electrocatalyst and the membrane are the central issues of MEA degradation. Concerning the loss of electrochemical surface area by Pt dissolution and reprecipitation phenomena of the Pt electrocatalyst during the operation, review papers are to be found based on many studies [10,11,12,13,14,15,16,17,18,19,20]. Additionally, there are some review papers of membrane degradation for practical applications [21,22,23]. In this paper, we focus on the chemical degradation of the membrane in MEA from an atomistic view to facilitate the understanding of the chemical reaction mechanisms of the membrane. 

## 2. Membrane Degradation in PFSA

Most of the membranes used in PEFC have a perfluorinated backbone and are modified to include sulfonic groups that facilitate the transport of protons. The requirements for an excellent membrane are high proton conductivity, as well as thermal and chemical stability. The commonly used membranes for PEFC are perfluorosulfonic acid (PFSA) polymers such as Nafion^®^ (Dupont), Gore-Select (Gore), Aciplex (Asaki Kasei), Flemion (AsahiGlass), and Celtec-P (BASF). The degradation of PFSA polymers accounts for a large part of the overall performance degradation of MEA. An understanding of the degradation mechanism of PFSA is thus important in the development of durable MEA. The degradation of PFSA can be classified into three categories, *i.e.*, mechanical, thermal, and chemical degradations. 

### 2.1. Mechanical Degradation

The mechanical degradation is caused by repetition of expansion and contraction associated with changes in wet and dry conditions. Long-term durability studies under various conditions have been reported in order to understand the performance decrease mechanism of PFSA under critical operating conditions [24,25,26,27,28,29,30,31,32,33,34]. Pozio *et al.*, reported that the long-term operation at low humidification led to a decrease of the three-dimensional reaction zone due to ionomer degradation by dehydration of the membranes. Schmittinger and Vahidi pointed out the importance of water management in membranes. To carefully analyze the water in the membrane, observation techniques of liquid water in the membrane were proposed by using time-resolved neutron radiography and high resolution dynamics in-plane neutron imaging [35,36,37,38]. In addition, the water distribution in the membrane was analyzed by computational fluid dynamics [39,40]. The mechanical degradation of the membrane becomes clear gradually on analysis of the distribution of liquid water and the performance of MEA. 

### 2.2. Thermal Degradation

The thermal degradation is caused by operations at freezing and high temperatures [41,42,43,44,45,46,47,48,49]. The most favorable working temperature of the PFSA membrane is usually around 80 °C to maintain a highly efficient operation. However, wide temperature range operation is required to use PEFC in various environments. Membrane degradation at sub-freezing temperature is one of the critical issues [41,42,43]. It has been observed that freezing water on the PFSA membrane leads to degradation due to different densities and conditions of water and ice. In addition, recently high temperature operation above 100 °C has been targeted for faster electrochemical kinetics, easier water management, and for the improvement of CO tolerance. The development of high durable and proton conductive membrane is one of the most important research fields. Recently, various kinds of membranes with blends, such as poly(arylene ether sulfonate ketone)s (SPESKs), sulfonated polyimide membrane containing triazole group (SPI-8), and sulfonated polyethersulfone (SPES), have been proposed for high durable membranes for thermal degradation [50,51,52,53,54,55,56,57,58,59]. 

### 2.3. Chemical Degradation

The chemical degradation is caused by the chemical reaction between membrane and chemical species. While mechanical and thermal degradation mechanisms can be understood macroscopically, atomistic understanding is essential for the chemical degradation mechanism. As a chemical species, it is generally believed that the degradation of PFSA membrane is caused by the attack of free radicals from hydrogen peroxide (H_2_O_2_) [60,61,62,63,64,65,66,67,68,69,70,71,72,73,74,75,76,77,78]. The H_2_O_2_ was confirmed in drain water, exhaust gas, and the membrane during operation of PEFCs. The H_2_O_2_ in PEFC is formed at the cathode from two-electron reduction of O_2_ by cross-leakage of O_2_ gas. The reactive hydroxyl (·OH) and hydroperoxide (·OOH) radicals are formed from H_2_O_2_. Recently, the hydrogen radical (·H) was also detected. Nosaka *et al.*, detected the OH radicals formed during the PEFC operation by a fluorescence probe method [62,63,65]. Although there are many experimental reports to analyze the OH formation mechanism [60,61,67,69,70,71,73], important knowledge is the chemical degradation of PFSA by radical species. This chemical degradation of PFSA is a microscopic phenomenon. It is necessary to analyze the chemical reaction between membrane and free radicals from an atomistic point of view. In the subsequent chapters, we summarize the recent studies clarifying the atomistic details of chemical degradation mechanisms of PFSA membrane using experimental and theoretical approaches.

## 3. Chemical Degradation Mechanism by Experiments

### 3.1. PFSA Main Chain

**Figure 1:**
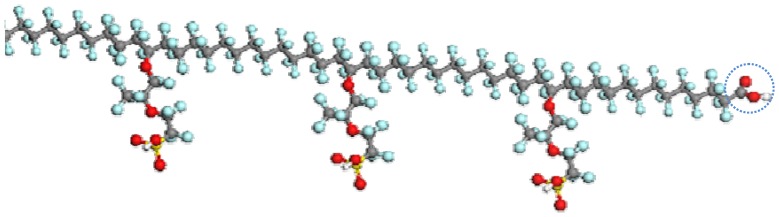
Structure of Nafion^®^ membrane.

Figure 1 shows the structure of PFSA polymer, which consists of a perfluorinated main chain and a side chain including sulfonic groups as well as ether and C-S bonds. 

The degradation mechanism of free radical and carboxyl end groups has been examined by using Fenton’s testing [79,80]. The chemical degradation of PFSA was explained by attack of the OH radical on the terminal –COOH group in the perfluorinated main chain. This unzipping mechanism is shown below [81].

Rf–CF_2_COOH + ·OH → Rf–CF_2_ + CO_2_ + H_2_O     (1)

Rf–CF_2_ + ·OH → Rf–CF_2_OH → Rf–COF· + HF     (2)

Rf–COH + H_2_O → Rf–COOH + HF         (3)

**Figure 2:**
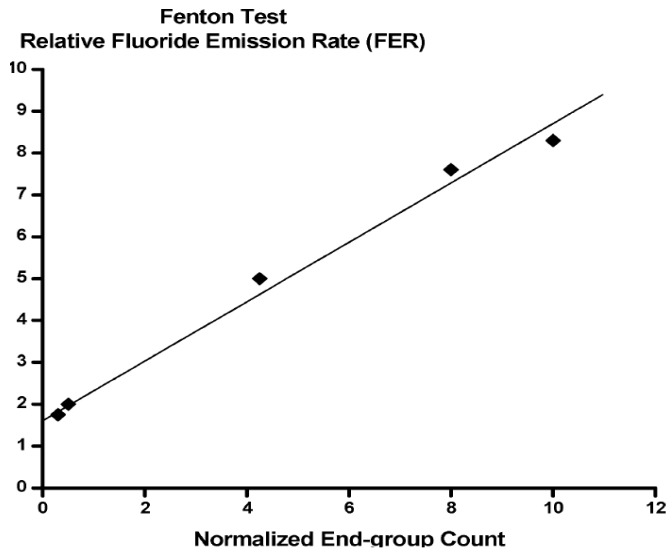
Plot showing relative fluoride emission rate (FER) from Fenton’s test as a function of concentration of reactive end-groups (taken from in reference [82]).

This mechanism indicates that the existence of the carboxylic (COOH) acid group in the perfluorinated main chain should be strongly related to the degradation of PFSA. This has been well-validated by plotting fluoride ion emission rate (FER) *vs.* carboxylic acid content in Nafion^®^ (Figure 2), however, a decidedly non-zero intercept can be seen in Figure 2. At an extrapolated value of zero carboxylic acid groups in Nafion^®^, over 10% of the total FER for untreated membrane remains [82,83]. This experimental result indicates the possibility of degradation by the attack of free radical except for the COOH group on the perfluorinated polymer main chain. Recently, the chemical degradation of the terminal carboxylic group in PFSA has not been the main issue of the chemical degradation, because several companies have addressed this problem by using original chemical stabilization procedures.

### 3.2. PFSA Side Chain

To explore the chemical degradation point in the PFSA membrane, Xie and Hayden analyzed the two possible PFSA chemical degradation mechanisms between the main chain end group and side chain [84]. From the IR spectra coupled with ionomer fluoride loss data, however, they found not only the carboxylic acid end group reaction of the main chain but also side chain cleavage reaction as PFSA chemical degradation pathways, whose contributions to overall degradation are difficult to quantitatively differentiate. To identify the degradation mechanism from the PFSA side chain, the chemical degradation and stability of PFSA polymer against OH radical attack were investigated by solid-state NMR, solution NMR, and IR spectro-electrochemical methods [85,86,87,88,89,90]. Although some chemical species were detected by these analyses, the degradation mechanism was still unclear. Ishihara *et al.*, synthesized the model compounds of the PFSA main and side chains, CF_3_(CF_2_)_8_SO_3_H and CF_3_(CF_2_)_3_O(CF_2_)_2_OCF_2_SO_3_H, respectively, to explore the chemical degradation in the PFSA side chain [91]. They measured oxidative degradation of model compounds under the following Fenton test conditions: 100 °C, 15% H_2_O_2_, catalyst FeSO_4_·7H_2_O, and 6–24 h. The result of the degradation experiment in aqueous solution is shown in Table 1. The model compound representing the PFSA side chain was not fully recovered, although the one representing the main chain was completely recovered. This result clearly indicates that the PFSA side chain is vulnerable to OH radical attack. Recently, Dreilzer and Roduner also pointed out that the major point of OH radical attack is ether groups of the PFSA side chains, determined by using ESR measurement and reaction kinetics analysis [92]. 

**Table 1:** Experimental result of oxidative degradation reaction (taken from reference [91]).

Model compounds	Temperature (°C)	Time (h)	Recovery (%)
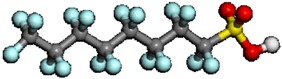	100	6	100
	100	24	100
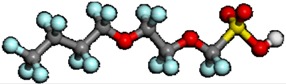	100	6	84
	100	24	80

## 4. Chemical Degradation Mechanism Studied by Theoretical Methods

### 4.1. Theoretical Approaches for PFSA Membrane

Computational simulation is one of the most effective approaches for understanding chemical and physical phenomena. Computational fluid dynamics and multi scale modeling are often used for thermal and transportation analyses in MEA and optimization of PEFC performance [93,94,95,96,97,98,99,100,101]. Although these approaches have advantages for the analysis of macroscopic phenomena, the analysis of microscopic phenomena is necessary to utilize different types of computational simulations. First-principles calculations contribute to the analyses of detailed chemical reaction mechanisms from an atomistic point of view. There are many theoretical reports about hydration around the PFSA membrane and proton conductivity in PFSA membrane [102,103,104,105,106,107,108,109]. In addition, the proton conductivity due to proton transfer in the membrane was analyzed [110,111,112,113,114,115,116,117,118]. Physical properties, such as morphology, of membrane were also analyzed [119,120,121,122,123,124,125]. These theoretical results found that the proton dissociation from the membrane depends on the number of water molecules around the sulfonic acid, which is the end group of the PFSA side chain. Deprotonation from the sulfonic acid to the water solution becomes favorable as the number of water molecules around the sulfonic acid group increases. This trend led us to assume that the protonated and deprotonated sulfonic acid structures correspond to two extremes, *i.e.*, low and high humidity conditions. This is important information to understand on the structure of PFSA polymer. The analysis from an atomic point of view by first-principles calculation is effective to aid understanding of the chemical degradation mechanism of PFSA membrane.

### 4.2. Bond Dissociation Analysis

**Figure 3:**
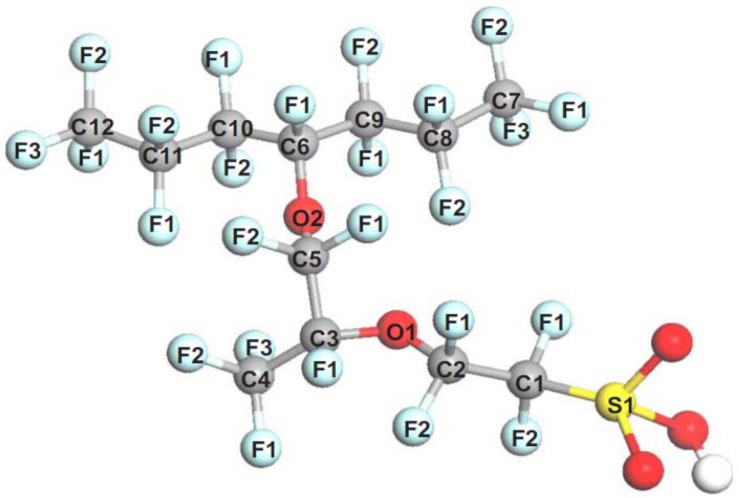
Molecular model of perfluorosulfonic acid (PFSA) polymer with atom labels (taken from reference [137]).

It is assumed that the degradation point of PFSA polymer by OH radical attack involves a relatively weak chemical bond in PFSA polymer. This information is useful in order to analyze the chemical degradation of PFSA polymer by OH radical attack based on the chemical reaction pathway analysis. One of the most useful applications is the analysis of bond dissociation energy. In fact, bond dissociation energy has been analyzed to understand the bond strength, bond character, and reactivity [126,127,128,129,130,131,132,133,134,135]. It was found that the C–F bond is relatively stronger than C–H. To identify the weak chemical bond in PFSA polymer, the estimation of bond dissociation energy in PFSA polymer is important. Coms carefully analyzed the C–F bond dissociation energies of various PFSA polymers containing C–O and C–S bonds [136]. He also analyzed the H and F abstraction energies by the OH radical. Based on these results of the thermochemical analysis, he confirmed the unzipping mechanism involving OH radical and pointed out the weak C–S bond including the PFSA side chain. Tokumasu *et al.*, analyzed the bond dissociation energy of the PFSA polymer side chain to provide some dissociation trends of the PFSA polymer [137]. The model structure they used is shown in Figure 3. 

**Table 2:** Bond dissociation energy of the side chain backbone in the PFSA model (kJ/mol). Labels denote the bonds shown in Figure 3.

Bond	Neutral	Ionized
S1–C1	257.3	349.8
C1–C2	356.5	411.4
C2–O1	342.7	383.4
O1–C3	337.6	299.1
C3–C4	332.8	338.1
C3–C5	332.4	349.6
C5–O2	306.2	328.4
O2–C6	290.3	222.0

The C–F bonds in the side chain showed that the bond dissociation energy decreases in the order of primary, secondary, and tertiary bonds. The C–S bond was the weakest in the side chain backbone in the neutral molecule, which is a model for low humidity condition. When ionized PFSA polymer structure was used, a model for high humidity condition, the C–S bond became stronger. On the other hand, the C–O bond in the ionized structure became weaker than that in the neutral. These results indicate the different chemical degradation mechanisms of PFSA polymer under low and high humidity conditions. In addition, the C–O bond might be a degradation point with OH radical attack.

### 4.3. Degradation by Chemical Reaction of PFSA Polymer and OH Radical

**Figure 4:**
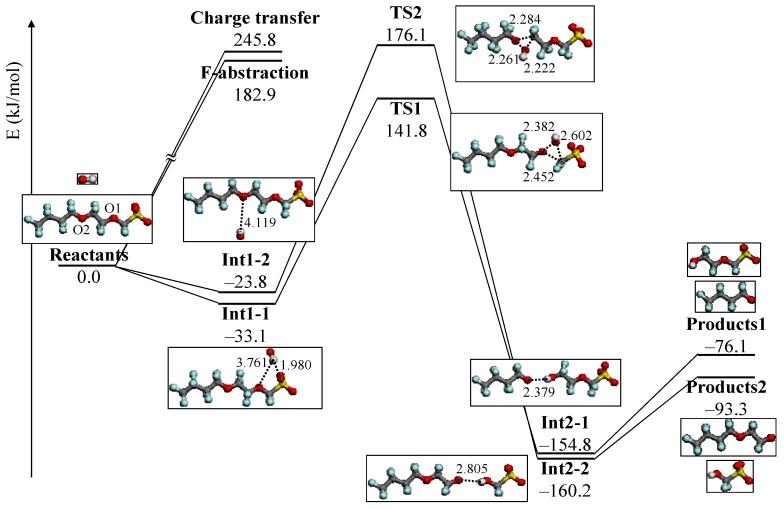
Potential energy profile under high humidity condition, CF_3_(CF_2_)_3_O(CF_2_)_2_OCF_2_SO_3_^−^ + OH. The energy values (kJ/mol) are relative to reactants. Optimized structures of reactants, products, intermediate, and transition state are also shown in the potential energy profile. Important distances are shown in Å.

**Figure 5:**
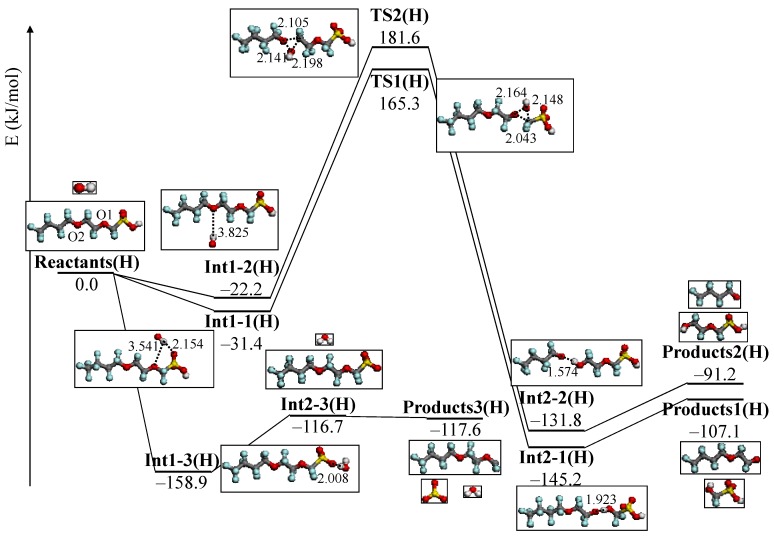
Potential energy profile under low humidity condition, CF_3_(CF_2_)_3_O(CF_2_)_2_OCF_2_SO_3_H +·OH. The energy values (kJ/mol) are relative to reactants. Optimized structures of reactants, products, intermediate, and transition state are also shown in the potential energy profile. Important distances are shown in Å.

Concerning the reactivity of the C–O bond by OH radical, there have been some studies with the first-principles calculation [138,139,140,141,142,143]. Based on these insights, we first theoretically analyzed the chemical degradation mechanism of the ether group in PFSA polymer model structures by the attack of OH radical [144]. We used the model structures CF_3_(CF_2_)_3_O(CF_2_)_2_OCF_2_SO_3_H representing the PFSA side chains because the experimental result by Ishihara *et al.*, suggested that the ether group in the PFSA side chain is vulnerable to OH radical attack. We performed a density functional theory (DFT) calculation to study the degradation reaction mechanism of the ether group in the model compound of the PFSA side chain with OH radical. The low and high humidity conditions of PFSA polymer side chain were represented by CF_3_(CF_2_)_3_O(CF_2_)_2_OCF_2_SO_3_H and CF_3_(CF_2_)_3_O(CF_2_)_2_OCF_2_SO_3_^−^, respectively. Figure 4 and Figure 5 show the potential energy profile under high and low humidity conditions, respectively. 

Under high humidity condition, we clearly demonstrated the degradation mechanism and reactivity of C–O bond cleavage in the ether group by the OH radical. This result shows reasonable agreement with the experimental one. However, the OH radical prefers the reaction with the sulfonic acid group under the low humidity condition. We found a different reactivity of the OH radical under high and low humidity conditions.

Furthermore, we analyzed the chemical reaction pathways of PFSA side chain and OH radical by DFT calculations to study the degradation mechanism of the PFSA side chain [145]. The proton dissociated Nafion^®^ was used as an example of PFSA polymer in the high humidity condition.

**Figure 6:**
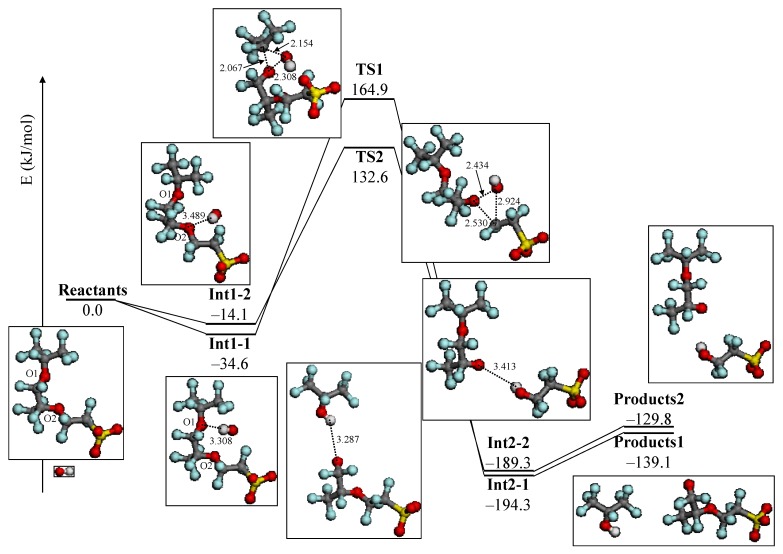
Potential energy profile under high humidity condition, model structure of Nafion side chain +·OH. The energy values (kJ/mol) are relative to reactants. Optimized structures of reactants, products, intermediate, and transition state are also shown in the potential energy profile. Important distances are shown in Å.

In this study, we calculated the reactivity between two ether groups in the Nafion^®^ side chain and OH radical. The potential energy profile of the reaction is shown in Figure 6. The reactions of O1 and O2 in the Nafion^®^ side chain attacked by the OH radical are denoted as reactions 1 and 2, respectively. Two reaction intermediate structures, Int1-1 and Int1-2, were obtained by weak attractive interaction between OH radical and O atoms in the ether groups. The stabilization energies of Int1-1 and Int1-2 were −34.6 and −14.1 kJ/mol, respectively. These intermediate compounds led to different products through the transition states. In the transition state, the C–O bond cleavage is induced by the OH radical in both cases. The energy of the C–O2 bond cleavage at TS2 was about 30 kJ/mol lower than that of C–O1 at TS1. We did not observe a difference in the chemical bond of the ether groups from the bond overlap population analyses. The atomic charges of O1 and O2 in the Nafion^®^ side chain were −0.473 and −0.478, respectively. Large stabilization was observed in Int1-1 because the O–H distance of Int1-1 is about 0.2 Å shorter than that of Int1-2. At the transition state, C–O1 and C–O2 bond distances in reactions 1 and 2 were 2.067 and 2.530 Å, respectively. This result indicates that the C–O2 bond cleavage at TS2 is favorable rather than C–O1 at TS1.

To discuss the difference of reactivity concerning the two ether groups with OH radical, we analyzed the ratio of the rate constants,

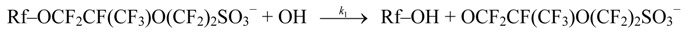
(4)

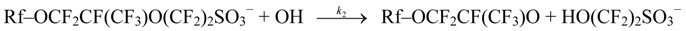
(5)
we evaluated the *k*_2_/*k*_1_ at 350 K using the following equation,

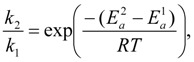
(6)
where the *R* and *T* are gas constant and temperature, respectively. The 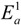 and 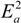 are activation energies of reactions (4) and (5), respectively. Because *k_2_*/*k_1_* is estimated as 4.0 × 10^3^, reaction (5) has a large advantage. We clearly demonstrated the degradation mechanism of the ether group in the Nafion^®^ side chain by the OH radical from theoretical analysis as well as from the previous results for Nafion^®^ model compounds. 

**Figure 7:**
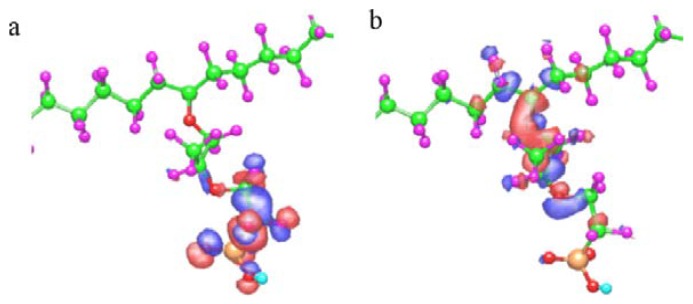
Optimized geometry of Nafion^®^ and their molecular orbitals: (**a**) highest occupied molecular orbital (HOMO) and (**b**) lowest unoccupied molecular orbital (LUMO) (taken from reference [146]).

Uegaki *et al.*, analyzed the highest occupied molecular orbital (HOMO) and lowest unoccupied molecular orbital (LUMO) based on frontier orbital theory [146]. These orbitals play an important role to estimate electrophilic and nucleophilic reactions. The HOMO and LUMO in Nafion^®^ are shown in Figure 7. The HOMO and LUMO are widely distributed around the terminal bond in the side chain and near main chain, respectively. They suggested that OH radical reacts around HOMO. Their expectation is similar to our analysis of the chemical degradation mechanism by C–O bond cleavage near the sulfonic acid group in the Nafion^®^ side chain with OH radical. They also expected the reactivity around LUMO, which is near the main chain, by O_2_^−^ and H· species.

Furthermore, Yu *et al.*, showed another chemical degradation mechanism of the Nafion^®^ side chain by OH radical [147]. They proposed two chemical mechanisms for OH radical attack on the Nafion^®^ polymer: (1) OH radical attack on the S–C bond to form H_2_SO_4 _plus a carbon radical followed by decomposition of the carbon radical to form an epoxide; (2) OH radical attack on H_2_ crossover gas to from a hydrogen radical, which subsequently attacks a C–F bond to form HF plus a carbon radical. This carbon radical can then decompose to form a ketone plus a carbon radical. The products (HF, OCF_2_, SCF_2_) from their proposed degradation mechanism have been observed by F NMR in the fuel cell [84]. 

**Figure 8:**
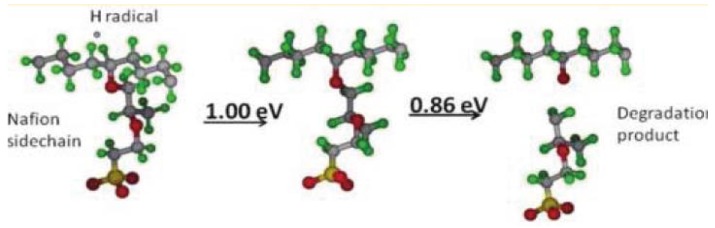
Degradation mechanism of Nafion^®^ proposed by Yu *et al.* (taken from reference [147]).

## 5. Summary

The degradation of PFSA can be classified into three categories, mechanical, thermal, and chemical. In this paper, we introduced recent studies on the chemical degradation of the PFSA membrane in MEA from an atomistic view. The chemical degradation is caused by the chemical reaction between the PFSA membrane and chemical species. As regards the chemical species, it is generally believed that the degradation of PFSA membrane is caused by the attack of free radicals, such as OH radical from hydrogen peroxide. The OH radical was experimentally detected and the formation mechanism of OH radical investigated. The chemical degradation of PFSA membrane by the attack of OH radical can be classified into two degradation points. One is from the carboxylic group of the main chain, which is referred to as an unzipping mechanism, the other is from the side chain. Some experimental results suggested that one of the possible degradation points of the PFSA side chain is the ether group. However, detailed analysis of chemical degradation is not easy using only experimental techniques. Recently, computational analysis from an atomistic point of view has been gradually introduced. The chemical bond strengths of the Nafion^®^ main and side chains were analyzed. The chemical degradation mechanism between the PFSA side chain and OH radical was rationalized by DFT calculations. The chemical degradation mechanism becomes clear from both molecular level experimental and theoretical analyses. After this breakthrough in the understanding of these chemical degradation mechanisms of the PFSA membrane, it is expected a durable membrane design of PEFC by combination of experimental and computational approaches will be achieved.
